# Exploration of the correlation between intestinal flora and *Escherichia coli* peritoneal dialysis-related peritonitis

**DOI:** 10.1186/s12882-022-02704-y

**Published:** 2022-02-22

**Authors:** Jun Zhou, Cuishun Yang, Wenjuan Lei, Zhen Yang, Jianmei Chen, Hua Lin, Qingtian Li, Wanqiong Yuan

**Affiliations:** 1 Department of Nephrology and Rheumatology, Haikou People’s Hospital Affiliated to Xiangya School of Medicine, Haikou, China; 2 Department of Nursing, Haikou People’s Hospital Affiliated to Xiangya School of Medicine, Haikou, China; 3 Department of Orthopedics, Guangdong Provincial People’s Hospital, Guangdong Academy of Medical Sciences, 106 Zhongshan ER Road, Guangzhou, 510080 China; 4 Department of Orthopedics, Peking University Third Hospital, 49 North Garden Rd., Haidian District, Beijing, 100191 China; 5 Beijing Key Laboratory of Spinal Diseases, Beijing, China; 6 Engineering Research Center of Bone and Joint Precision Medicine, Ministry of Education, Beijing, China

**Keywords:** *Escherichia coli*, Gut microbiota, Gut dysbiosis, Peritonitis

## Abstract

**Background:**

*Escherichia coli* peritonitis (EP) is a serious complication of peritoneal dialysis (PD). Gut microbiota alterations occur in end-stage renal disease (ESRD) patients. The relationship between the gut microbiota and PD-related peritonitis is still poorly understood. It is unclear whether the intestinal flora is involved in the pathogenesis of EP.

**Methods:**

We collected fecal samples from EP patients and normal group (NG) PD patients. 16S rRNA sequencing was used to analyze the gut microbiota of EP and NG patients. The demographic data and clinical indicators of all patients were collected.

**Results:**

Six EP patients and 28 NG patients were recruited for this study. The analysis of fecal community diversity with 16S rDNA sequencing showed an obvious change in the microbial structure of EP patients, where Bacteroidetes and Synergistetes were upregulated at different levels, while Bacilli and Lactobacillus were downregulated at different levels compared to the NG group. Additionally, decreased gene function associated with metabolic pathways was observed in EP patients.

**Conclusions:**

The altered composition of the gut microbiota in EP patients provided deeper insights into the pathogenesis of EP, and these biomarkers might be established as potential therapeutic targets that deserve further exploration.

## Background

Peritonitis is one of the major complications of peritoneal dialysis (PD) and remains the primary reason that patients switch from peritoneal dialysis to hemodialysis [1]. Mainly due to improvements in the connectology of PD made during recent decades, peritonitis caused by gram-positive pathogens in PD has decreased markedly [2]. However, the incidence of gram-negative peritonitis has not decreased to the same extent, and its proportion has consequently increased [3].

Enterococcus and Enterobacter are the most common organisms that cause enterogenous peritonitis in PD patients [4]. *Enterococcus faecium* and *Enterococcus faecalis* account for the leading proportion of enterococcus [4], and the *Enterobacter* spp. are mainly *Escherichia coli* and *Klebsiella* [5]. Previous studies have shown that constipation, diarrhea, hypokalemia and hypoalbuminemia are significantly associated with *Escherichia coli* peritonitis (EP) [6]. These findings highlight the importance of intestinal flora in EP.

Currently, disturbances of the normal gut microbiome are associated with the pathogenesis of several chronic diseases, including chronic kidney disease (CKD) [7]. Intestinal disorders is well described in CKD, characterized by lower levels of Bifidobacteriaceae and Lactobacillaceae and higher levels of Enterobacteriaceae [8]. Renal replacement therapy (RRT) used in patients with end-stage renal disease (ESRD) also affects the composition of the microbiome. Micro ecological imbalance is more obvious in hemodialysis (HD) patients, where increases in potentially pathogenic species and decreases in beneficial species are often observed in patients. This dysbiosis is also present in PD patients [9]. A study has revealed that the peritoneal microbiota plays a key role in promoting infection or progression in ESRD patients, especially PD patients [10]. However, the relationship between the gut microbiota and peritonitis is still poorly understood. It is unclear whether the intestinal flora is involved in the pathogenesis of EP. In the present study, the species differences were analyzed between the EP group and the non peritonitis patients with PD group by the 16S rRNA amplification sequencing method. The composition and diversity of the intestinal flora in EP group were determined. This study lays a foundation for further studying the relationship between the intestinal flora and peritonitis in EP patients.

## Methods

### Patients

Peritoneal dialysis patients were enrolled from Haikou People’s Hospital between September 2019 and October 2020. Fecal samples were collected before antibiotic treatment. An EP group and the control group were formed. The normal control group (NG) was as the control group. Inclusion criteria were: 1) The PD patients without peritonitis at all during time on PD; 2) patients over 18 years of age; 3) continuous peritoneal dialysis treatment for more than 3 months, stable clinical condition during observational period; 4) informed consent was signed. PD patients with severe hepatitis, cirrhosis, tumors and tuberculosis were excluded. Antibiotics, preparations of live bacteria, lactulose and other drugs were prohibited 2 weeks before sampling [1].

### Study definitions

EP-related peritonitis was diagnosed based on three of the following criteria [11]: (1) abdominal pain or cloudiness of PD effluent; (2) white blood cell count in PD effluent > 100/μl with > 50% polymorphonuclear leukocytes; and (3) *Escherichia coli*-positive culture from PD effluent. There are at least 1 of the above first two items, and the third items is the necessary condition.

### Laboratory test

The demographic data and clinical indicators of all patients were collected. In the EP group, these indicators were collected on admission. In the NG, the indicators were collected before fluid exchange in the fasted state.

### Microbiology test

For microbiology tests, 50 mL PD effluent (PDE) was centrifuged at 3000 g for 15 min, and then the pellet was inoculated in Bact/Alet anaerobic and aerobic bottles (BioMerieux, Durham, NC, USA). The identities of all isolates and their susceptibilities to certain antibiotics were determined using the Vitek-2 Auto Microbic system (BioMerieux, St. Louis, MO, USA).

### Fecal sample collection and storage

Fecal specimens were naturally collected before antibiotic treatment. Fresh stool samples cannot be analyzed immediately and need to be preserved for a while. Thus, fecal materials were instantly frozen at − 80 °C.

### DNA extraction from fecal samples

DNA from different samples was extracted using the E.Z.N.A.®Stool DNA Kit (D4015, Omega, Inc., USA) according to the manufacturer’s instructions. The reagent, which was designed to uncover DNA from trace amounts of sample, has been shown to be effective for the preparation of DNA of most bacteria. Nuclear-free water was used as a blank. The total DNA was eluted in 50 μL of elution buffer and stored at − 80 °C until measurement by PCR by LC-Bio Technology Co., Ltd., Hang Zhou, Zhejiang Province, China.

### PCR amplification and 16S rDNA sequencing

In this study, the V3-V4 [12] region of 16S rRNA was amplified using the forward primer (5′-CCTACGGGNGGCWGCAG-3′) and reverse primer 805R (5′-GACTACHVGGGTATCTAATCC-3′); primers for the V4 region were [13]:515F (5′-GTGYCAGCMGCCGCGGTAA-3′), 806R (5′-GGACTACHVGGGTWTCTAAT-3′); primers for the V4-V5 region were F (5′-GTGCCAGCMGCCGCGG-3′), R (5′-CCGTCAATTCMTTTRAGTTT-3′); and those for Archae were [14]: F (5′-GYGCASCAGKCGMGAAW-3′), R (5′-GGACTACHVGGGTWTCTAAT-3′]. Amplification was performed in a total volume of 25 μL of reaction mixture containing 25 ng of template DNA, 12.5 μL of PCR Premix, and 2 μL of each primer]. The PCR conditions to amplify the prokaryotic 16S fragments consisted of an initial denaturation at 98 °C for 30 s; 32 cycles of denaturation at 98 °C for 10 s, annealing at 54 °C for 30 s, and extension at 72 °C for 45 s; and then a final extension at 72 °C for 10 min. The PCR products were confirmed with 2% agarose gel electrophoresis. Throughout the DNA extraction process, ultrapure water, instead of a sample solution, was used as a negative control to exclude the possibility of false-positive PCR results. The PCR products were purified by AMPure XT beads (Beckman Coulter Genomics, Danvers, MA, USA) and quantified by Qubit (Invitrogen, USA). The amplicon pools were prepared for sequencing, and the size and quantity of the amplicon library were assessed on an Agilent 2100 Bioanalyzer (Agilent, USA) and with the Library Quantification Kit for Illumina (Kapa Biosciences, Woburn, MA, USA), respectively. The libraries were sequenced on the NovaSeq PE250 platform.

### Data analyses

Samples were sequenced on an Illumina NovaSeq platform according to the manufacturer’s recommendations, provided by LC-Bio. Paired-end reads were assigned to samples based on their unique barcodes and truncated by cutting off the barcode and primer sequence. Paired-end reads were merged using FLASH. Quality filtering of the raw reads was performed under specific filtering conditions to obtain high-quality clean tags according to fqtrim (v0.94). Chimeric sequences were filtered using Vsearch software (v2.3.4). After dereplication using DADA2, we obtained a feature table and feature sequence. Alpha diversity and beta diversity were calculated by normalization to the same sequences randomly. Then, according to the SILVA (release 132) classifier, feature abundance was normalized using the relative abundance of each sample. Alpha diversity was applied to analyze the complexity of species diversity for a sample through 5 indices, including Chao1, observed species, Good’s coverage, Shannon, and Simpson indices, and all these indices for our samples were calculated with QIIME2. Beta diversity was calculated by QIIME2, and the graphs were drawn by the R package. Blast was used for sequence alignment, and the feature sequences were annotated with the SILVA database for each representative sequence. Other diagrams were implemented using the R package (v3.5.2) [15].

### Statistical analysis

Data analysis was performed using the SPSS 23.0 software package. The measurement data were expressed as the mean ± SD and were compared by t-test. The nonnormal data were expressed as medians and were compared by rank sum test. The Wilcoxon rank-sum test or Kruskal-Wallis test were used for diversity differential analysis, depending on the different groups. *P* < 0.05 was considered statistically significant.

## Results

### Participant characteristics

Six EP patients and 28 NG patients were recruited between September 2019 and October 2020. In the EP group, there were 4 males and 2 females. The demographic and clinical characteristics of the patients are shown in Table 1.

**Table 1:** Demographic and clinical characteristics of patients who underwent *E. coli* peritonitis (*n* = 6) and the nonperitonitis normal group (*n* = 28)

Characteristics	NG	EP	Value
Age (years)	48.14 ± 14.26	42.67 ± 16.48	0.412
Gender (male/female)	22/6	4/2	0.454
Etiology of ESRD [n, (%)]			0.083
Glomerulonephritis	19	2	
Diabetic nephrology	6	2	
Urinary calculus	1	1	
Other/Unknown	2	1	
PD vintage (months)	40 ± 28.44	34.83 ± 20.41	0.667
Serum potassium (mmol/l)	3.61 ± 0.88	3.58 ± 0.59	0.905
Albumin (g/l)	28.16 ± 5.11	26.96 ± 2.61	0.523
White blood cells (×109/l)	7.56 ± 3.15	10.24 ± 3.24	0.038
Neutrophil percentage	0.71 ± 0.09	0.85 ± 0.06	0.001
C-reactive protein (g/l)	18.44 ± 24.8	74.04 ± 58.5	0.000

### The diversity and taxa of the fecal microbiota

**Fig. 1:**
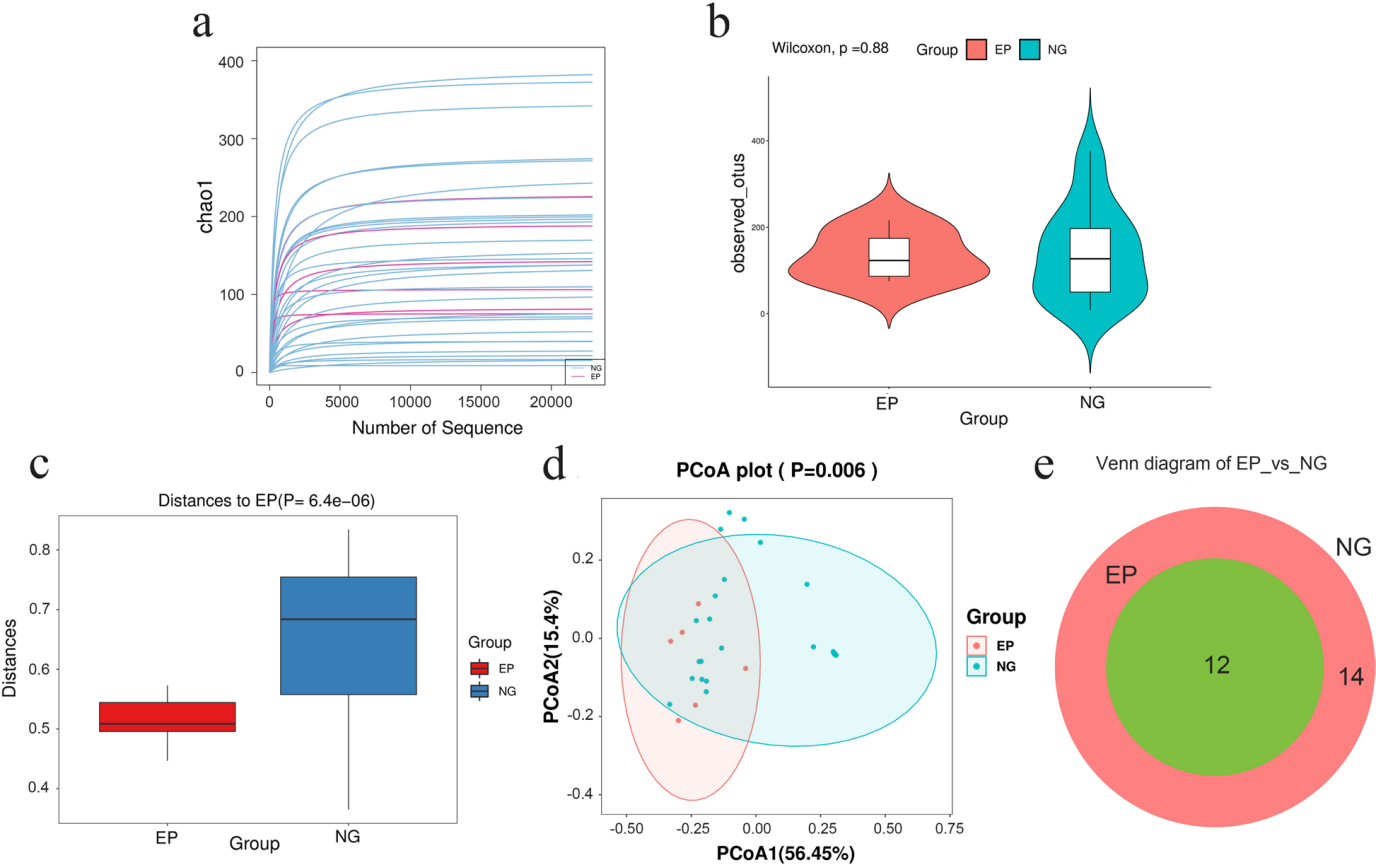
Preliminary comparison of the diversity of gut microbiota between the EP group and the NG group. **a** The quantities and differences of ASVs among the samples are directly shown in the rarefaction curves of the Chao1 index, which tend to flatten and suggest the sufficiency and reasonableness of the sequences. **b** The boxplot of the Shannon index shows that there was no significant difference in ASV diversity between the EP group and NG (*p* = 0.88). **c** Analysis of similarities (ANOSIM) (*p* < 0.001) indicated that the between-sample discrepancies in microbial structure were significant. **d** Principal coordinate analysis (PCoA) with weighted UniFrac distances (*p* = 0.006) shows that the distance between two points indicates a significant difference in community composition. PCoA1 and PCoA2 are used to plot the coordinate axis, and the percentages refer to the extent of variation explained. **e** The Venn diagram intuitively presents the number of common and exclusive ASVs between the EP group and the NG group calculated using R software

The sparse curve of the Chao1 index showed that all the curves tended to be smooth and reach a plateau, which indicates that the amount of sequencing data was sufficient and rational and that more data would only generate a small number of new ASVs (Fig. 1a). In addition, there was no significant difference in richness and evenness of microorganisms between the EP and controls, as showed in the Shannon index box chart (*P* = 0.88, Fig. 1b). Moreover, ANOSIM also showed that significant differences in the intestinal microbiota structure between the EP group and NG group (*P* < 0.001, Fig. 1c). There were differences between two groups in weighted UniFrac PCoA (*P* = 0.006, Fig. 1d). Nevertheless, the Venn diagram (Fig. 1e) exhibited differences and visually stated the exact number of ASVs unique (12 in the EP group and 14 in the controls) to the groups (Fig. 1e). These data preliminarily elucidated the significant changes in intestinal microflora structure in the EP group.

**Fig. 2:**
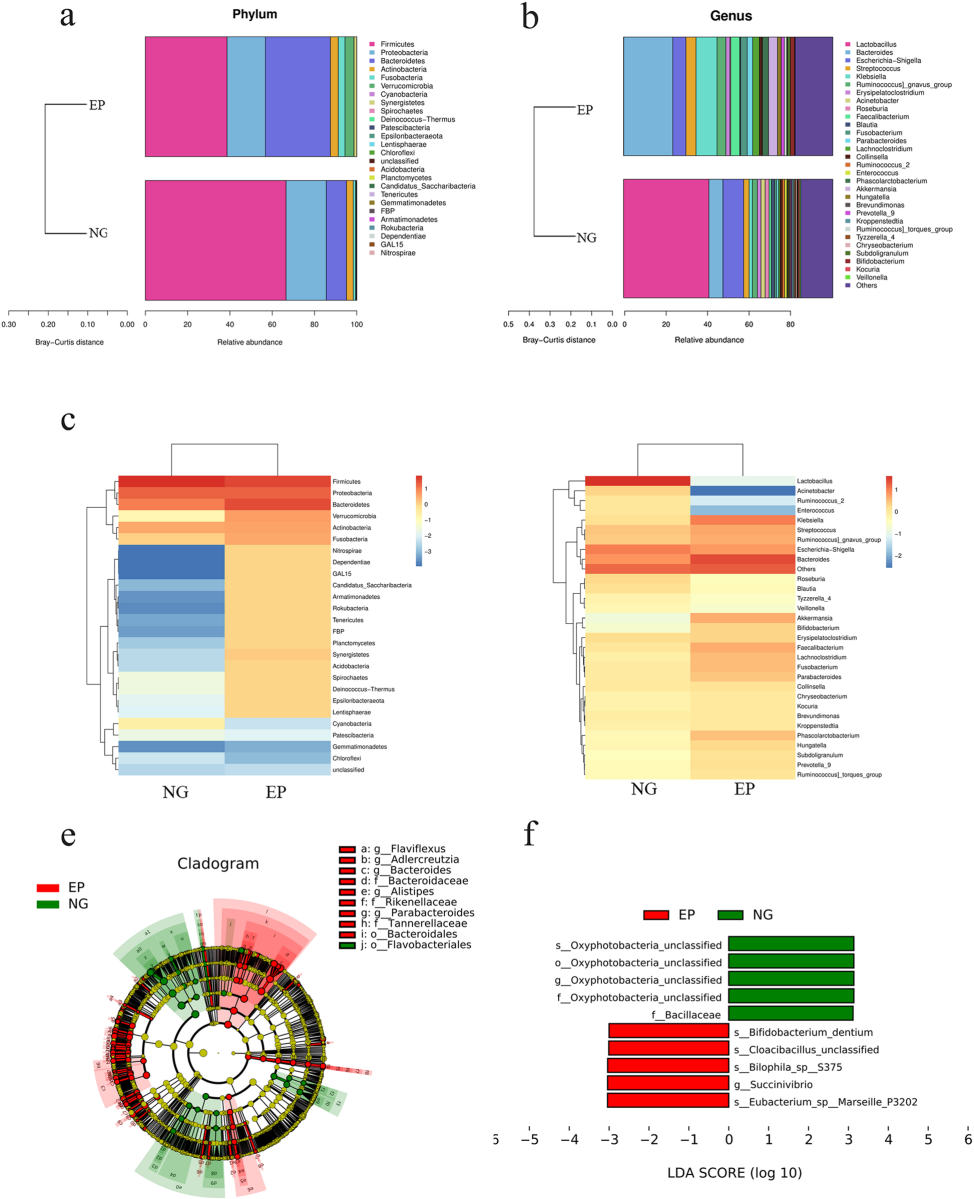
Taxonomic characterization of gut microbes and biomarkers in the EP group and NG. **a**, **b** The taxonomic distributions and relative abundance of the fecal microorganisms from both groups are shown in stacked bar charts at the phylum level and the genus level (only top 30 displayed). Different colors represent different microbes at the same level. The bar from bottom to top corresponds to the relative abundance from high to low. **c**, **d** The taxa heatmap reveals the similarities and differences between the EP group and the NG group at the phylum level and the genus level (only the top 30 displayed) on the basis of the Bray–Curtis distance through 16S rDNA sequencing. The abundance profiles were transformed into Z scores by subtracting the average abundance and dividing the standard deviation of all samples, using the signal sample (log2) to map the original value. The color gradient from blue to red is used to reflect the abundance from low to high. **e** The cladogram results of LEfSe show taxonomic clades that are differential in abundance, where differential circles from the inside to the outside represent different classification levels from domain to species and the larger size of the nodes reflects higher relative abundance. The yellow nodes indicate no significant differences, and the biomarkers are colored red and green depending on the group. **f** The bar chart shows the biomarkers with differential abundance between the groups and those higher than the preset value (LDA score > 4, *p* < 0.01). The LDA score indicates the extent to which the corresponding group is affected by the differential microbes

**Fig. 3:**
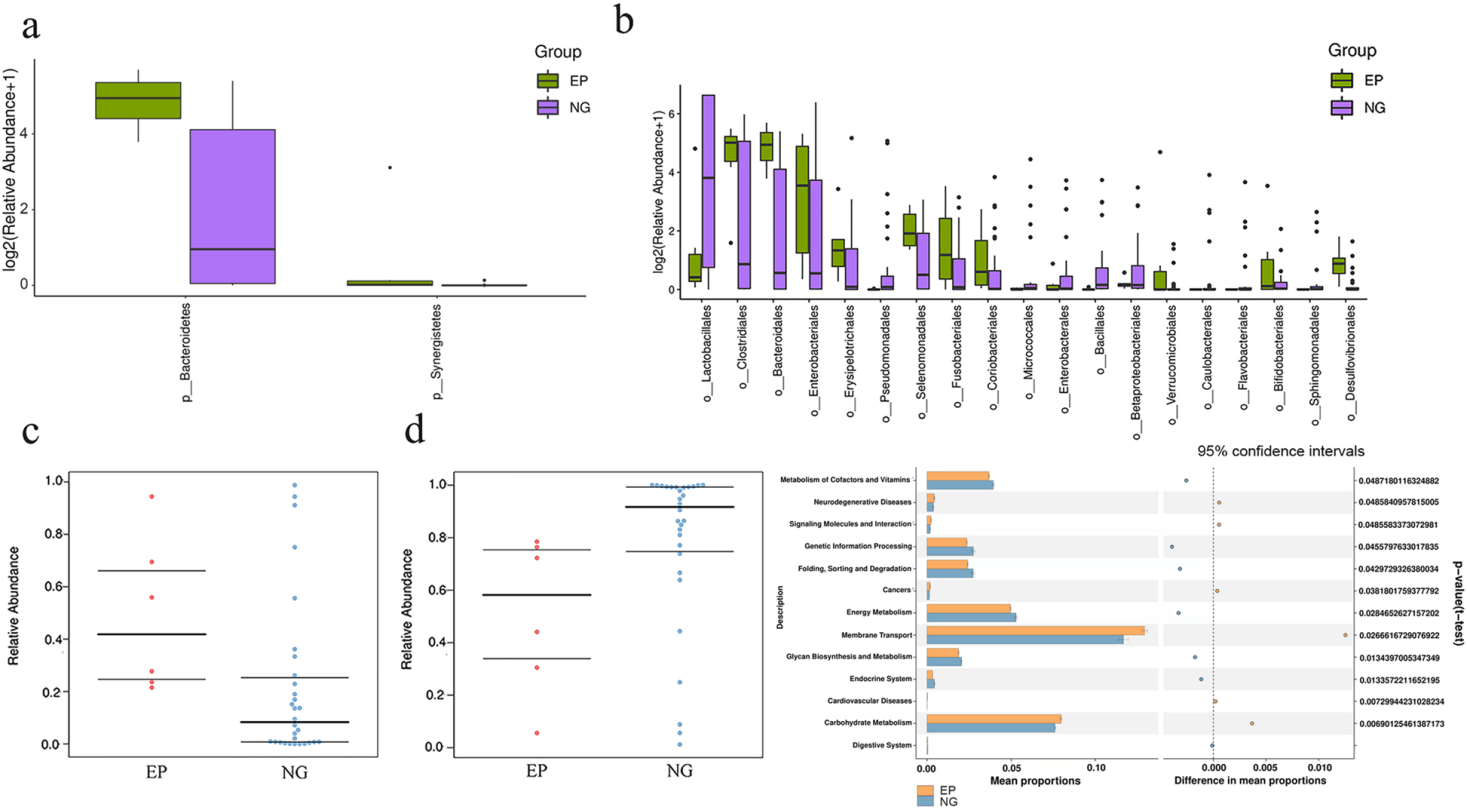
**a** Bacteroidetes and Synergistetes were significantly increased in the EP group at the phylum level (*p* < 0.05) (**b**). Bacillales and Lactobacillales were significantly decreased in the EP group at the order level (*p* < 0.05). **c** Phenotype prediction showed that Gram-negative status was significantly increased in the EP group (*P* = 0.013). **d** Phenotype prediction showed that the Gram-positive status was significantly decreased in the EP group (*P* = 0.013). **e** The KEGG pathway analysis of the second level was compared with metabolic analysis between the EP group and the NG. (1). For the left half of the graph, the Y axis represents the KEGG pathways of levels. The length of the column indicates the KO abundance in the pathway. (2). For the right half of the graph, the X axis represents the confidence interval of the difference in species abundance, and the Y axis represents the *P* value. KEGG: Kyoto encyclopedia of genes and genomes

To further characterize the intestinal microbiota associated with EP, we annotated the ASVs with both the Silva and NT-16S databases, producing a large number of microorganisms at the domain, phylum, class, order, family, genus and species levels. The fecal microbiota based on Bray–Curtis distance are distributed in the stacked bar charts (Fig. 2a, b), where the relative abundance of Bacteroidetes (30.78 vs. 9.58%, *P* = 0.01) and Synergistetes (1.3 vs 0%, *p* = 0.00) increased significantly in the EP group at the phylum level (Fig. 3a). On the contrary, Firmicutes (38.64 vs. 66.54%, *P* = 0.07) and Proteobacteria (18.12 vs 19.04%, *p* = 0.7) were reduced in the EP group. The annotation chart showed only the 30 most abundant microbes at the genus level, and therefore did not exactly correspond to the altered microbes. Furthermore, the distribution of rich intestinal communities was mapped with a taxa heatmap (Fig. 2c, d). In addition, according to LEfSe (LDA score > 3, *P* < 0.05), Bacteroidetes at the phylum level (LDA = 5.02, *P* = 0.01), Bacteroidia at the class level (LDA = 5.02, *P* = 0.01), Bacteroidales at the order level (LDA = 5.03, *P* = 0.00) (Fig. 3b), Bacteroidaceae at the family level (LDA = 4.91, *P* = 0.01) and Bacteroides at the genus level (LDA = 4.91, *P* = 0.01) were higher in the EP group and could be used as biomarkers to predict the risk of EP (Fig. 2e, f). In addition, Synergistetes in the EP group at the phylum level (LDA = 3.76, *P* = 0.00), Synergistia at the class (LDA = 3.79, *P* = 0.00), Synergistales at the order (LDA = 3.78, *P* = 0.00) (Fig. 3b), Synergistaceae at the family (LDA = 3.79, *P* = 0.00), and Pyramidobacter at the genus level (LDA = 3.8, *P* = 0.03) were also higher than those in the EP group. In comparison, Bacilli at the class level (LDA = 5.35, *P* = 0.03), Bacillales at the order level (LDA = 3.84, *P* = 0.01) (Fig. 3b), and Bacillaceae at the family level (LDA = 3.12, *P* = 0.01) were all lower in the EP group. In addition, Lactobacillales at the order level (LDA = 5.34, *P* = 0.03) (Fig. 3b), Lactobacillaceae at the family level (LDA = 5.33, *P* = 0.01) and Lactobacillus at the genus level (LDA = 4.91, *P* = 0.03) were also lower in the EP group. Taken together, these results suggested that the intestinal microbiome composition of EP patients was indeed affected.

### Phenotype prediction

The prediction of potential phenotypic functions of bacteria in the two groups of different types of samples revealed nine potential microbial phenotypes including aerobic, anaerobic, mobile element-containing, biofilm-forming, facultative-anaerobic, Gram-negative, Gram-positive, potentially pathogenic, and stress tolerant phenotypes (Fig. 3c, d). The differences in the relative abundance of two predicted phenotypic functions (Gram-negative and Gram-positive) were significant (*P* < 0.05), while the rest were not significant (*P* > 0.05).

### Functional annotation prediction

To reveal the potential distinction in gene function between the EP group and controls, metagenomics was carried out. PICRUSt2 software was utilized to predict the gene function of the fecal microbiome via the KEGG pathway library, and we annotated 40 different KEGG pathways (Fig. 3e) at level 2. Thirteen significantly different KEGG pathways were identified in these two groups at level 2 (Fig. 3e). There was a significant decrease in pathways involved in the digestive system (*P* = 0.01), energy metabolism, folding, sorting and degradation (*P* = 0.04), and glycan biosynthesis and metabolism (*P* = 0.01) in the EP group compared to the NG group. Thus, our data exhibited the differentially expressed functions annotated using the KEGG databases, suggesting that the metabolic patterns in EP patients might be distinctive from those in NG controls.

## Discussion

In this study, we found that the WBC count, neutrophil percentage, and CRP were significantly increased in the EP group compared to those in the NG (*p* < 0.05). The levels of ALB and serum potassium were low in the two groups. Hypokalemia and hypoalbuminemia may ultimately lead to a reduction in normal gut flora, which ferment fiber to produce short-chain fatty acids (SCFAs), which act as a key energy source for colorectal tissues and symbiotic microbes [16]. However, ALB and serum potassium were not significantly decreased (*p* > 0.05) between the two groups. This finding is not in line with previous studies showing that hypokalemia and hypoalbuminemia were significantly associated with EP. Hypokalemia and hypoproteinaemia are common complications in PD patients due to the dietary restriction of potassium and potassium and protein being lost from the fluid. In addition, this may be related to the peritonitis patients having been in the hospital without obvious gastrointestinal symptoms. However, the sample size was small in the EP group.

In the present study, the alpha diversity of the gut microbiota of each group showed no significant difference based on the ACE, observed species, or Shannon indices, which indicated that there was no difference in the flora diversity. In addition, the saturated rarefaction curve indicated that the sequence number of the samples was sufficient for the analysis. The beta diversity of the gut microbiota in the two groups was evaluated based on the unweighted UniFrac distance. The results showed that there were significant differences distinguishing the two groups by the weighted UniFrac distances. This indicates that disturbance in the EP patient gut microbiome may currently be associated with the pathogenesis of EP.

This study also found that the intestinal flora of EP patients in comparison to NG patients was significantly different at the phylum, class, order, family, genus, and species levels. At the phylum, class, order, family and genus levels, EP patients exhibited significantly increased Bacteroidetes and decreased Firmicutes, while Proteobacteria and Actinobacteria showed no difference between the two groups. The ratio of Firmicutes to Bacteroidetes decreased in our study. A study showed that Actinomycetes, Firmicutes and Proteobacteria were the dominant orders in ESRD patients [17]. Vaziri’s group found that Actinobacteria, Firmicutes (especially Clostridium) and Proteobacteria were markedly increased in patients with HD in comparison to healthy controls [18]. Wang’s group found that Firmicutes and Actinomycetes were decreased, especially probiotics such as *Bifidobacterium longum*, *Lactobacillus plantarum* and *Lactobacillus parabases* [19]. In present study, the pathogenesis of significantly increased Bacteroidetes in EP patients was unclear. Because EP and ulcerative colitis (UC) have similar changes in the gut microbiota. At present the change of Bacteroides abundance in patients with UC was controversial. Bacteroides is a common intestinal symbiotic anaerobe, which can cause endogenous infection when the immune function is disturbed or the intestinal flora is disturbed [20]. Then we can speculate significantly increased Bacteroidetes can cause endogenous infection in EP patients. More research is needed to explain the potential role of changes in Bacteroides in EP patients.

In this study, we also found Bacilli at the class level, Bacillales at the order level, and Bacillaceae at the family level, while the order Lactobacillales, family Lactobacillaceae and genus Lactobacillus significantly decreased in the EP group. Thus, our results were consistent with those of these studies. We postulated that the ratio of Firmicutes to Bacteroidetes could be a potential biomarker of EP.

More studies have shown that an increase in Enterobacteriaceae members is one of the reasons for peritonitis in PD patients [21]. Lactobacillus is the dominant microbiome in the human intestinal tract and is especially abundant from the duodenum to the terminal ileum. Lactobacillus are bacteria that can ferment carbohydrates to produce large amounts of lactic acid. The adhesion of Lactobacillus to the intestinal mucosa can maintain the integrity of the intestinal mucosa and plays an important role in ensuring intestinal flora homeostasis [22]. Butyric acid is the main metabolite of butyrate, one of the main SCFAs [23]. Hu reported that PD reduced microbial diversity, decreased probiotic butyrate-producing microbiota and increased urease, indole, and p-cresol-forming microbiota [24]. A study demonstrated that long dialysis duration, high peritoneal glucose exposure, and loss of residual renal function were associated with gut microbiota alteration and reduced branched-chain SCFA production in PD patients [23]. The gut microbiota producing SCFAs is decreased, which impairs the intestinal epithelial barrier structure and function, thereby facilitating the translocation of intestinal microorganisms, endotoxins, antigens and other microbial products through the intestinal wall toward the systemic circulatory system and the internal milieu [25]. This may easily lead to peritonitis for PD patients. Stadlbauer et al. found that gut microbiome dysbiosis was more pronounced in HD patients and was associated with an increase in CRP but not with intestinal inflammation or gut permeability [26]. However, the relationship between the gut microbiota and peritonitis is still poorly understood. In recent years, the relationship between PD patients and the gut microbiota has been investigated to determine possible therapeutic targets (prebiotics and probiotics, such as p-inulin) to improve patient quality of life and improve survival for ESRD [27].

The results of bacterial functional prediction and microbiome phenotype prediction showed that the gram-negative status was significantly increased in the EP group (*P* = 0.013), while the gram-positive status was significantly decreased. This indicates that bacteria in different groups have different functions and phenotypes. EP patients are more susceptible to gram-negative infection.

Data on alterations in metabolism-related signaling pathways were derived by PICRUSt analysis. Gut microbial functional prediction showed that the EP group had significantly decreased pathways such as Digestive System, Energy Metabolism and, Glycan Biosynthesis and Metabolism. These findings are consistent with the results of abundance analysis and species diversity. The increased Bacteroidetes in the EP group could metabolize plant polysaccharides that cannot be digested and absorbed by the host for 10–15% more energy [28]. This is an adaptation to changes in the intestinal environment; however, when the capacity cannot meet the demand for energy, it may lead to an imbalance in energy metabolism and eventually develop into protein-energy waste.

Our study had several shortcomings. First, although our data showed that the intestinal flora changed at different classification levels, however the sample size was relatively limited; therefore, further studies with larger sample numbers are needed. Second, all of subjects were recruited from a center that shared a limited geographical area and could be biased in terms of participant diet. Our study did not assess patient diets and drug intake; therefore, the influence of these factors on our study data cannot be excluded. Third, the fecal samples of patients were not collected many times, which could better reflect the dynamic changes of the intestinal flora. Further studies are needed to elucidate the effect of PD treatment on the fecal microbiome of EP patients.

In conclusion, EP remains a serious complication of PD. Our results indicate that disturbance in the EP patient gut microbiome is linked with a higher risk of developing peritonitis. The decreasing ratio of Firmicutes to Bacteroidetes could be a potential biomarker of EP. The altered composition of the gut microbiota in EP patients provided deeper insights into the pathogenesis of EP. Of note, our human data are observational, and the number of subjects was relatively low, so these data are not sufficient for the development of specific treatment recommendations. However, our work to some extent lays a foundation for larger follow-up studies that might validate the causal effect of the gut microbiome and even generate a new therapy.

## Data Availability

The datasets generated and analyzed during the current study are available from the corresponding author on reasonable request.

## Abbreviations

EP: *Escherichia coli* peritonitis
PD: Peritoneal dialysis
ESRD: End-stage renal disease
NG: The normal control group
CRP: C-reaction protein
ALB: Albumin
CKD: Chronic kidney disease
RRT: Renal replacement therapy
HD: Hemodialysis
PDE: PD effluent
PCoA: Principal coordinate analysis
LEfSe: Linear discriminant analysis effect size
PICRUSt: Phylogenetic investigation of communities by reconstruction of unobserved states
KEGG: Kyoto Encyclopedia of Genes and Genomes
SCFA: Short chain fatty acids
ASVs: Amplicon single variants
ANOSIM: Analysis of similarities
